# Volumetric brain alterations in children with primary complex motor stereotypies: a baseline and longitudinal report

**DOI:** 10.3389/fneur.2026.1800595

**Published:** 2026-07-22

**Authors:** Megan E. Markiewicz, Deana Crocetti, Cade C. Mills, Farhan Augustine, Alyssa C. DeRonda, Stewart H. Mostofsky, Harvey S. Singer

**Affiliations:** 1 Center for Neurodevelopmental and Imaging Research, Kennedy Krieger Institute, Baltimore, MD, United States; 2 Department of Neurology, The Johns Hopkins University School of Medicine, Baltimore, MD, United States; 3 University of Colorado Anschutz Medical Campus, School of Medicine, Aurora, CO, United States; 4 Department of Biological Sciences, University of Maryland Baltimore County, Baltimore, MD, United States; 5 Department of Neurology and Department of Psychiatry and Behavioral Sciences, The Johns Hopkins University School of Medicine, Baltimore, MD, United States; 6 Division of Neurology, Kennedy Krieger Institute, Baltimore, MD, United States

**Keywords:** primary complex motor stereotypies, pathophysiology, MR volumetric measurements, frontal cortex, basal ganglia, cerebellum

## Abstract

**Background:**

Primary complex motor stereotypies (pCMS) are early-onset, repetitive movements in otherwise typically developing children. Although symptoms often improve during adolescence, neuroanatomical mechanisms underlying the pathophysiology of movements and symptom reduction remails unclear. Structural MRI studies have implicated multiple structures within cortical-basal ganglia-thalamo-cortical circuits, but findings have been inconsistent and limited by small, cross-sectional childhood cohorts. It remains unclear whether reported volumetric differences represent static markers of pCMS or developmental changes related to clinical severity.

**Methods:**

Volumetric MRI examined frontal cortical, basal ganglia, and cerebellar regional volumes in participants with pCMS and typically developing (TD) controls at childhood (8–12 years) and adolescence/young adulthood (15–23 years). Cross-sectional analyses evaluated diagnostic effects on regional volumes (childhood: pCMS *n* = 33, TD *n* = 33; adolescence: pCMS *n* = 12, TD *n* = 9) and clinical severity correlations in pCMS (childhood *n* = 31, adolescence *n* = 10). Longitudinal analyses in pCMS evaluated whether changes in regional volumes from childhood to adolescence/young adulthood correlated with changes in stereotypy severity (*n* = 10).

**Results:**

Cross-sectional analyses in childhood revealed smaller left putamen volume and an association of smaller medial prefrontal volume with greater clinical severity in pCMS (neither finding survived FDR correction). Cerebellar analyses demonstrated larger anterior vermis gray matter and smaller anterior and posterior white matter volumes (surviving FDR correction); furthermore, larger cerebellar volumes were nominally associated with greater clinical severity (not surviving FDR correction). In adolescence/young adulthood, no frontal lobe or basal ganglia differences were detected. Cerebellar analyses identified nominally smaller anterior white matter volumes and associations between larger posterior white matter volumes and greater clinical severity (neither finding survived FDR correction). Longitudinal analyses revealed within-subject increases in frontal lobe gray matter volumes (predominantly right hemisphere), was associated with increased pCMS severity (not surviving FDR correction).

**Conclusions:**

Findings suggest that pCMS may be associated with regionally specific and developmentally dynamic structural differences rather than a single neuroanatomical abnormality. Childhood cerebellar sensorimotor regions demonstrated volumetric differences, while additional cross-sectional and longitudinal findings raise the possibility that frontal cortical, basal ganglia, and cerebellar regions may contribute differentially to stereotypy severity across development. Collectively, these findings are consistent with, but do not establish a circuit-level framework for understanding pCMS.

## 1 Introduction

Complex motor stereotypies (CMS) are non-goal directed (purposeless and non-pathological), recurrent (continuously repeated), rhythmic (moving in a particular rhythm), predictable (having a similar form), and distractible (stopping with distraction). Commonly observed examples of these complex movements include bilateral flapping, waving, or rotating movements of the arms and hands, fluttering fingers in front of the face, opening and closing of the hands, and finger wiggling. Movements typically begin before age 3 years, diminish in intensity in the teenage-years (1), last for seconds to minutes, and occur multiple times per day often in association with periods of excitement, stress, fatigue, or visual imagery (2–7). CMS is classified into two groups: primary, indicating an otherwise developmentally typical child; and secondary, for those with developmental disorders (e.g., autism, Rett syndrome, Lesch-Nyhan syndrome), sensory conditions such as deafness and blindness, and other metabolic or genetic disorders (8, 9).

**Figure 1:**
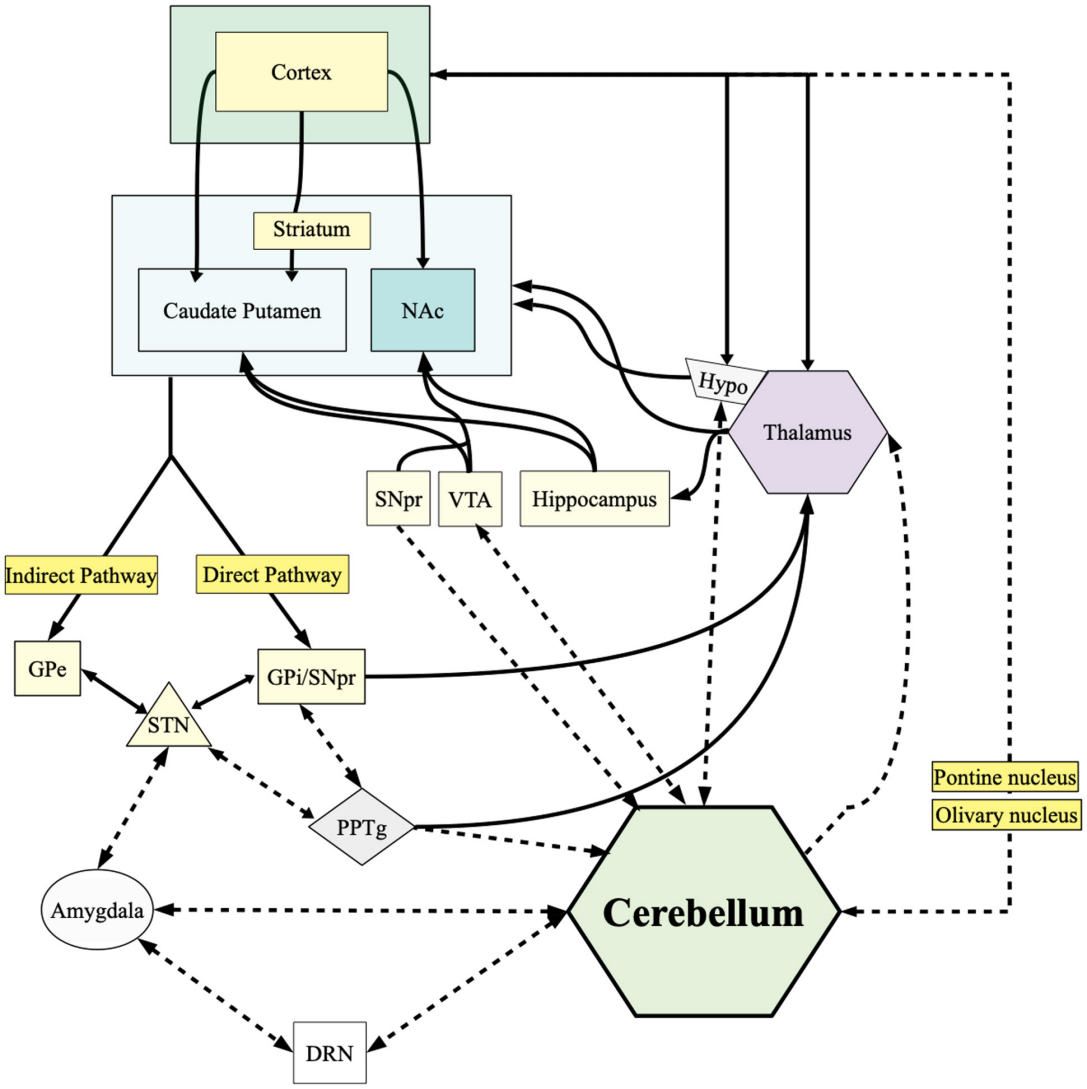
Interactions between cerebellum and CBGTC circuitry. Adapted from Singer and Augustine (57).

The precise etiology and anatomical brain region involved in the pathophysiology of primary CMS (pCMS) remains unknown. Being an abnormality of motor control, stereotypies have often been broadly localized to the cortical-basal ganglia-thalamo-cortical (CBGTC) circuitry (Figure 1), a complex network of pathways originating from neuronal networks within in the cerebral cortex (principally frontal cortex), with projections to the basal ganglia and cerebellum, and ultimately back to the cerebral cortex via the thalamus (10). Although the CBGTC circuit offers an intuitively appealing framework, it oversimplifies complex neurobiology by neglecting the regional functional specialization within these circuits and their extensive integration with other brain systems, including regions of the ventral striatum (motivation and reward), cerebellum (motor coordination), hippocampus (motor memory), amygdala (emotional and motivational modulation of movement), and dopaminergic nuclei such as the substantia nigra and ventral tegmental area, which regulate reward and learning (11). Recognizing the continued scientific need to identify specific neuroanatomical alterations in children with pCMS, this study utilized expanded methodologies to re-investigate volumetric abnormalities previously reported in several small magnetic resonance neuroimaging studies, with a focus on proposed alterations within frontal-cortical, basal ganglia, and cerebellar regions.

Prior volumetric studies have included 1.5 and 3.0 Tesla protocols comparing small populations of children with pCMS to typically developing controls. Results to date, suggesting volumetric reductions in frontal white matter and the caudate (12); the right putamen and a trend for reduction in the left putamen volume (13); and in the cerebellum a 10% increase in the anterior vermal gray matter that positively correlated with motor stereotypy severity and a significant reduction in posterior cerebellar lobules VI-VII that correlated with motor control but not stereotypy severity (14). Unfortunately, the current existing literature, summarized above, is notably limited to small cross-sectional studies conducted exclusively in young (school-age) children with pCMS. In contrast, the present study, with volumetric focuses on the basal ganglia, frontal cortex, and cerebellum, includes two separate cross-sectional analyses comparing individuals with pCMS to typically developing (TD) controls; one in younger children (Time Point 1) and a second in adolescents/young adults (Time Point 2). In addition, a separate two-point longitudinal analysis was conducted to examine whether changes in regional brain volumes were associated with changes in stereotypy severity.

## 2 Materials and methods

Approval for this study was granted by the Johns Hopkins Medicine Institutional Review Board. Written informed consent was obtained from a parent, or legal guardian, for participants under 18 years of age, with assent obtained from minors. Participants aged 18 years or older provided their own written informed consent.

### 2.1 Diagnostic criteria

All subjects were initially recruited from either the Johns Hopkins or Kennedy Krieger Institute (KKI) Pediatric Movement Disorder Clinics where they were formally evaluated and diagnosed by a child neurologist with expertise in pediatric movement disorders. Visual confirmation of stereotypies was obtained either by direct observation in clinic or via parent provided video. All participants had the onset of motor stereotypies before age 3 years, with persistent core features including bilateral arm and hand movements (flapping, waving, or rotation) or finger fluttering in front of the face. Other common features of the motor repertoire included occurrence during periods of engrossment, excitement, stress, fatigue or boredom, a prolonged duration, and suppression with distraction. Inter-individual variability was observed in the developmental sequence and duration of movements, as well as in the presence/absence of comorbid diagnoses.

Exclusion criteria for children in both the pCMS and TD control groups included a current full-scale intelligence quotient (FSIQ) of less than 80 as measured by the Wechsler Intelligence Scale for Children Fourth Edition (WISC-IV) (15) or Fifth Edition (WISC-V) (16); a diagnosis of autism spectrum disorder (ASD); presence of visual impairment, hearing loss ≥ 25 dB, or neurological disorder (e.g., epilepsy, cerebral palsy, and traumatic brain injury); treatment with psychotropic medication in the past three months; or a medical contraindication to MRI procedures. No participant had a history of a severe chronic medical disorder, diagnosed genetic disease, or psychotic disorder. Children with a first-degree relative with ASD or a parent-reported history of developmental or psychiatric disorders were excluded.

### 2.2 Participants

Participants included 33 children (18 boys and 15 girls) with pCMS who had an initial MRI scan (Time Point 1) between the age of 8.01–12.7 years; (Mean = 9.36; SD = 1.06) and 33 TD controls, 18 boys and 15 girls, ages 8.12–12.04 (Mean= 9.31, SD = 0.91). Additionally, 12 of the original pCMS participants (6 boys and 6 girls) returned for a second visit (Time Point 2) between the age of 15.76 and 23.44 years (Mean = 19.38, SD = 2.29) after a mean time interval of 10.15 years (SD = 2.49) from their initial visit. The follow-up visit included a repeat MRI scan and an evaluation of clinical severity. TD controls matched with the pCMS participants in the Time Point 1 cohort did not return for a follow-up visit. Therefore, a separate group of TD controls including 6 boys and 3 girls, ages 15.22–23.48 (mean age = 18.91), SD = 2.72, obtained from ongoing studies at the KKI Center for Neurodevelopmental and Imaging Research (CNIR), were included as a comparison group for the Time Point 2 cohort (Table 1).

**Table 1:** Participant characteristics.

	Time point 1			Time point 2		
	pCMS *n* = 33	TD *n* = 33	*p*-value	pCMS n = 12	TD *n* = 9	*p*-value
**Age**	9.36 (1.06)	9.31 (0.90)	0.851	19.38	18.91	0.680
**Sex (M:F)**	18:16	18:16	1.00	6:6	6:3	0.445
**SES**	56.02 (8.26)	51.37 (13.63)	0.111	–	53.17 (2.93)	–
**FSIQ**	106.42 (14.79)	110.70 (12.98)	0.217	105.42 (10.53)	116.44 (16.29)	0.100
**Head Coil (8ch:32ch)**	33:0	27:6	**0.010**	0:12	1:8	0.237
**TCV (SE)**	1,052,232.65 (16,751.19)	1,043,155.40 (1,6751.19)	0.711	1,008,562.46 (35,510.46)	1,033,279.97 (42,355.82)	0.691
**SSS total motor score**	10.84 (2.58)	–	–	6.40 (4.72)	–	–

Data are the mean (SD) unless otherwise stated. Significant *p-values* are indicated in bold. pCMS, primary complex motor stereotypy; TD, typically developing; SES, social economic status derived from the hollingshead; not collected for CMS follow-up appointment; FSIQ, full scale intelligence questionnaire derived from WISC IV, WISC V, and WASI II; TCV, total cerebral volume, stereotypy severity derived from the stereotypy severity scale (SSS) only in CMS participants.

### 2.3 Clinical assessments

The severity of stereotypy symptoms was rated on the day of the MRI scan using the Stereotypy Severity Scale (SSS) Parent Report (17). The SSS has two components (Motor and Impairment) for the ranking of motor stereotypy severity. The Motor score (range 0–18) quantifies motor severity and rates movements in four discriminate dimensions including: number (0–3), frequency (0–5), intensity (0–5), and interference (0–5). The SSS Impairment score (0–50) measures difficulties in self-esteem, family, school, or social acceptance caused by the movements during the past few days. For the purposes of the analyses performed in this paper, we focused on the SSS Motor Score.

### 2.4 Imaging

Structural MRIs were acquired at the F.M. Kirby Research Center for Functional Brain Imaging at KKI. A high-resolution T1-weighted 3D MP-RAGE image covering the whole brain was acquired for each participant on a Philips 3T MRI scanner (Best, the Netherlands) using either an 8 or 32-channel head coil (TR = 8 ms; TE = 3.76 ms, FOV = 25.6 cm, flip angle = 8°, voxel size = 1 mm isotropic). Qualitative quality assessment was performed by visual inspection, whereby each MP-RAGE image was classified into one of 5 categories, with 1 being “good” and 5 being “poor,” based on the degree and presence of distortion, ringing, ghosting, positional shifts, signal dropout, or blurring. Quantitative quality assessment was performed using the Image Quality Ratio (IQR) from the CAT12 (18) toolbox. MP-RAGE scans with an IQR below 70% were excluded. See Supplement Figure 1 for an illustration of the regional brain segmentation methods detailed below.

#### 2.4.1 Frontal (prefrontal, premotor, motor) cortex

*FreeSurfer* (19) was used for cortical reconstruction and volumetric segmentation of the frontal lobe using a pediatric-based atlas developed by the CNIR (20). Regions of interest (ROIs) included 10 subdivisions the dorsolateral pre-frontal cortex (dLPFC), medial pre-frontal cortex (mPFC), inferior lateral prefrontal cortex (ilPFC), lateral orbitofrontal cortex (lOFC), medial orbitofrontal cortex (mOFC), premotor (frontal eye field (FEF), lateral premotor cortex, supplementary motor complex (SMC), anterior cingulate cortex (ACC), and the primary motor cortex (M1). Frontal ROIs were comprised of gray matter only and were examined separately for each hemisphere. *FreeSurfer* was also used to generate total cerebral volume (TCV) which was subsequently used for covariate analyses. The ROIs were examined separately for each hemisphere and included 20 regions in total.

#### 2.4.2 Basal ganglia: caudate, putamen, and globus pallidus

MRICloud (www.mricloud.org), a web-based platform for automated hierarchical segmentation, was used for delineation of basal ganglia structures for volumetric analysis. The segmentation pipeline was built upon the diffeomorphic multi-atlas likelihood-fusion (MALF) (21) algorithm that leverages information from multiple atlases. In this work, the atlases were comprised of 30 brains (22), manually delineated by expert raters at the CNIR. Basal ganglia regions included the caudate, putamen, and globus pallidus and were examined separately for each hemisphere and included 6 regions in total.

#### 2.4.3 Cerebellum

Cerebellar segmentation was based on a pediatric cerebellar atlas developed at the CNIR (23). The cerebellar atlas is based on a highly reliable manual parcellation protocol with interclass correlation coefficients ranging from 0.86 to 0.99 across eleven anatomically defined subdivisions including the corpus medullare, hemisphere lobules I–V, VI, crus I, crus II/VIIB, VIII, IX, and X, and vermis lobules I–V, VI–VII, and VIII–X.

Regional cerebellar volumes were derived using Automatic Cerebellum Anatomical Parcellation using U-Net with Locally Constrained Optimization (ACAPULCO), a convolutional neural network (CNN) based cerebellar parcellation method (24). ACAPULCO derived CNNs were generated using the manual pediatric cerebellar parcellations described above. Cerebellar parcellation classifiers were trained from 20 pediatric manual delineations. See prior publications for further details (23, 25). Gray and white matter tissue was classified using SPM12 (26). Cerebellar regions were examined separately for each tissue type (gray and white matter) and hemisphere, with exception of the vermis regions, which were not split by hemisphere. Within tissue type analyses included 17 gray matter regions and 18 white matter regions.

### 2.5 Statistical analysis

Group differences in demographic characteristics and intellectual functioning were assessed using independent-samples *t* tests for age, social economic status (SES), and FSIQ. Sex and head coil distribution were assessed using a chi-square test.

Group differences in TCV at Time Point 1 and Time Point 2 were examined using univariate two-tailed analyses of variance (ANOVA) with significance at *p* < 0.05. Longitudinal change in TCV was assessed using mixed linear effects models.

To account for multiple comparisons across regions of interest, the Benjamini–Hochberg false discovery rate (FDR) (27) procedure was applied across all ROIs within each model using a significance threshold of α = 0.05.

#### 2.5.1 Cross-sectional effect of diagnosis on brain volume

Multivariate general linear models using two-tailed tests examined the impact of stereotypy diagnosis on regional frontal, basal ganglia, and cerebellar volumes with significance at *p* < 0.05 for each time point. Effect sizes were reported for all models to estimate the magnitude of group differences independent of statistical significance and were quantified using partial eta squared ($\eta_{p}^{2}$). Effect sizes were interpreted according to conventional benchmarks (small ≈ 0.01, moderate ≈ 0.06, large ≈ 0.14). All cross-sectional models were adjusted for TCV, FSIQ, and head coil. All analyses were performed using SPSS v31. This analysis was performed cross sectionally in the Time Point 1 cohort (ages 8–12, pCMS *n* = 33, TD *n* = 33) and in the Time Point 2 cohort (ages 15–23, pCMS *n* = 12, TD *n* = 9).

#### 2.5.2 pCMS Brain Volume and SSS Correlations

Pearson two-tailed partial correlations examined the association between regional frontal, basal ganglia, and cerebellar volumes and the SSS Motor Score for the pCMS participants only as the SSS was not collected from TD controls. All models were adjusted for TCV and FSIQ. Within the pCMS group, head coil was the same across participants at each time point; all Time Point 1 participants were scanned using an 8ch head coil and all Time Point 2 participants were scanned using a 32ch head coil. Therefore, coil was not included as covariate in these models. Benjamini and Hochberg FDR corrections were used to correct for multiple comparisons at α = 0.05. This analysis was performed cross sectionally in the Time Point 1 cohort (ages 8–12 years, pCMS *n* = 31) and in the Time Point 2 cohort (ages 15–23 years, pCMS *n* = 10).

#### 2.5.3 Developmental changes in pCMS brain volume and stereotypy severity

Longitudinal associations between stereotypy severity and regional brain volumes were examined using linear mixed-effects models to account for repeated measurements within subjects. Models included a random intercept for subject to account for within-subject dependence across time points. Models were estimated using restricted maximum likelihood (REML).

To examine whether changes in regional brain volume were associated with changes in symptom severity, brain volume was decomposed into within-subject and between-subject components. For each region of interest (ROI), the subject-specific mean volume across time points was calculated, and the time-varying deviation from this mean was entered into the model as the within-subject volume term. This approach isolates the association between individual changes in brain volume and concurrent changes in clinical severity, independent of stable between-subject differences in brain structure.

The primary model was specified as:


$$\begin{array}{l} Stereotypy\;Severity_{ij}=\beta_{0}+\beta_{1}\left(Time_{ij}\right)\\ +\beta_{2}\left(TotalCerebralVolume_{ij}\right)+\beta_{3}\left(FSIQ_{ij}\right)\\ +\beta_{4}\left(RegionalVolume_{dev,ij}\right)+\beta_{5}\left(RegionalVolume_{mean,i}\right)\\ +u_{0i}+\epsilon_{ij} \end{array}$$

where *i* indexes subject and *j* indexes time point. The coefficient for the within-subject volume deviation term represents the association between longitudinal changes in brain volume and changes in severity. *Positive coefficients therefore indicate that at time points when individuals exhibited higher-than-usual volume in a given region, the SSS Motor Score was also elevated*.

Models included TCV and FSIQ as covariates to account for individual differences in global brain size and cognitive ability. Continuous predictors were mean centered prior to analysis. The models were fitted using the GAMLj v3.0.0 (28) module in Jamovi (29), which utilizes the lme4 (30) for estimation. Given the modest sample size, bootstrapped confidence intervals (*n* = 1,000) for fixed effects were used to estimate parameter uncertainty. The models included 19 observations from 10 participants. Significance was defined as *p* < 0.05 (two-tailed).

Within the pCMS cohort, different head coils were used at Time Point 1 (8ch) and Time Point 2 (32ch), resulting in perfect confounding between coil and time. Therefore, coil could not be included as a covariate, and longitudinal effects may partially reflect coil differences. This limitation is acknowledged in the interpretation of the results.

## 3 Results

Age did not differ significantly between the pCMS and TD groups at either Time Point 1 (*p* = 0.851) or Time Point 2 (*p* = 0.680). There were no significant differences in sex between the pCMS and TD groups at either Time Point 1 (*p* = 1.00) or Time Point 2 (*p* = 0.445). SES did not differ significantly between groups at Time Point 1 (*p* = 0.111) and was not collected in pCMS participants at Time Point 2. FSIQ did not differ significantly between groups at Time Point 1 (*p* = 0.217) or at Time Point 2 (*p* = 0.100). Head coil distribution differed significantly at Time Point 1 (*p* = 0.010). All 33 participants in the pCMS group were scanned using an 8-channel head coil, whereas participants in the TD group were scanned using a mix of 8- and 32-channel head coils (27 and 6 participants, respectively). At Time Point 2, head coil distribution did not differ significantly between groups (*p* = 0.237). All 12 pCMS participants were scanned using a 32-channel head coil, and most TD participants were scanned using a 32-channel head coil (8 of 9 participants). TCV did not differ significantly between the pCMS and TD groups at Time Point 1 (*p* = 0.711) or Time Point 2 (*p* = 0.691). Within the pCMS group no significant effect of time (Time Point 2 minus Time Point 1) was observed for TCV (β = −23,128.0, SE = 12,175, *t* = −1.90, *p* = 0.098). See Table 1.

**Figure 2:**
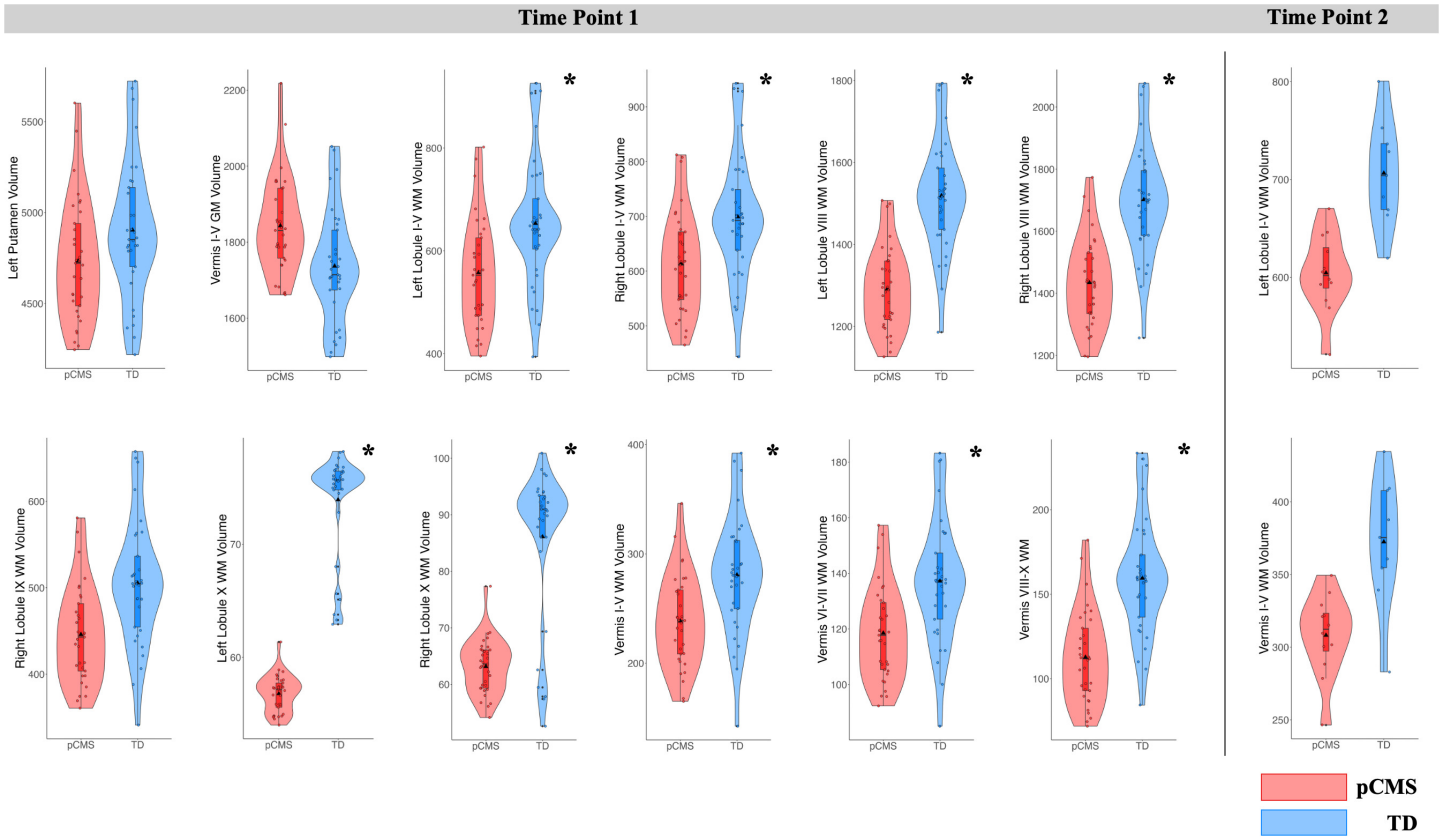
Distribution of regional brain volumes in the pCMS and TD groups shown using box-violin plots. Only anatomical regions demonstrating a significant main effect of diagnosis are displayed. Asterisks indicate effects that remain significant after false discovery rate (FDR) correction for multiple comparisons (α = 0.05).

**Figure 3:**
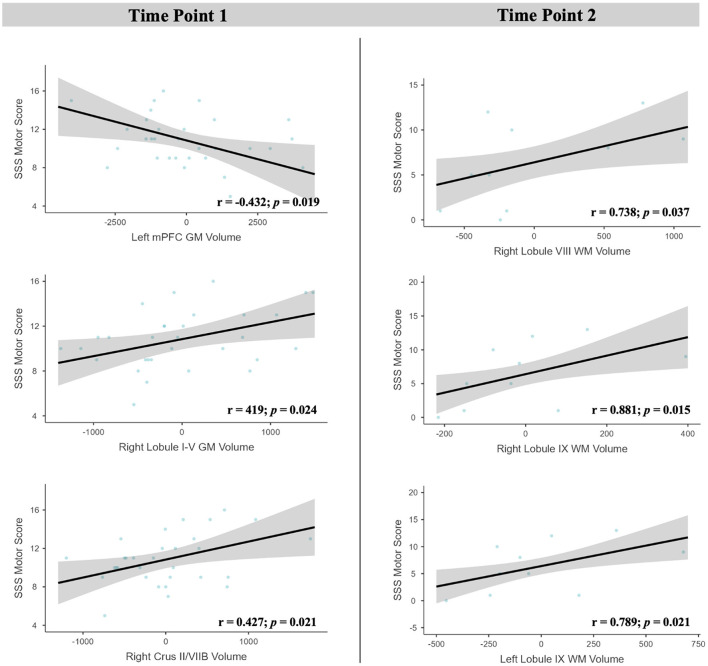
Scatterplots showing how regional brain volumes relate to stereotypy severity scores in the pCMS group at two time points. Pearson correlation coefficients are shown for regions where larger or smaller brain volumes were associated with symptom severity. None of these associations remained significant after FDR correction for multiple comparisons (α = 0.05).

**Figure 4:**
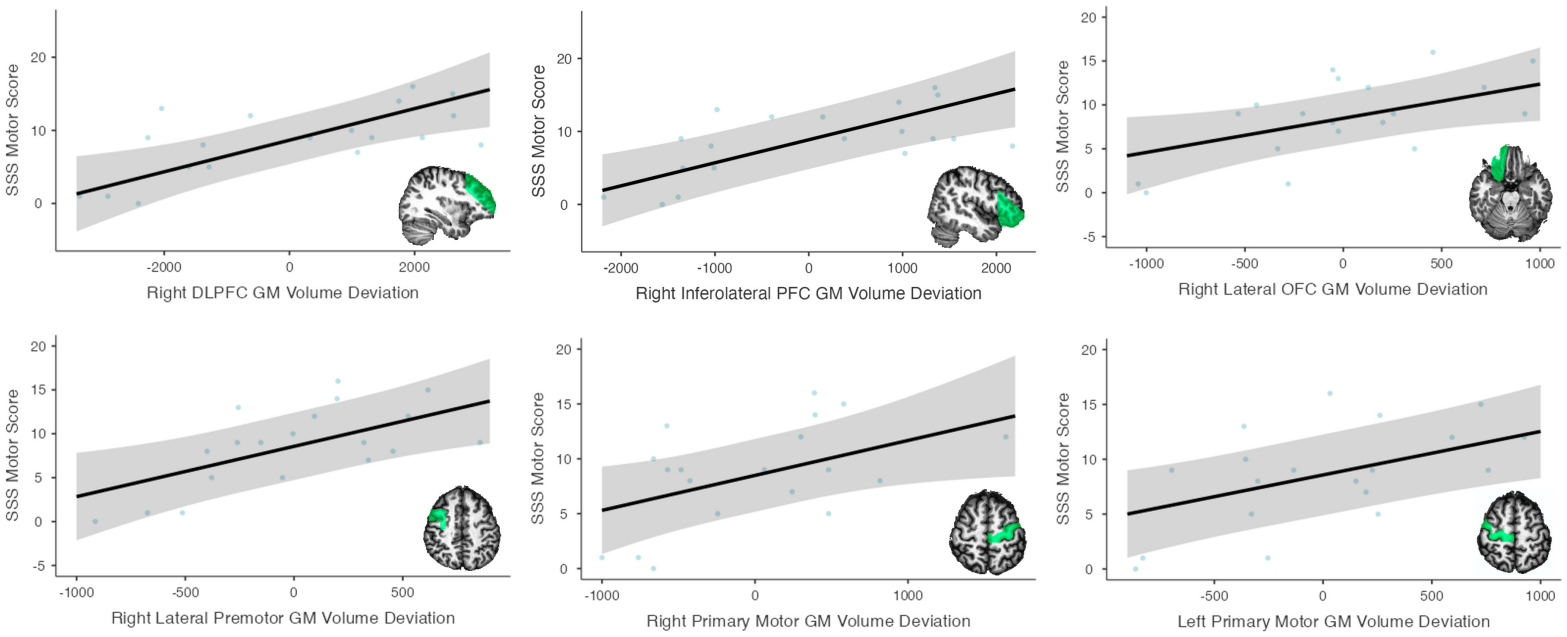
Within-subject associations between frontal lobe gray matter volume deviations and clinical severity. Scatterplots display significant within-subject effects from linear mixed-effects model examining the association between time-specific deviations in regional frontal lobe gray matter volume and SSS motor scores across visits in the pCMS group only. The models used within- and between-subject decomposition to separate time-varying effects from stable individual differences. Volume deviation was calculated as each participant's regional volume at a given time point minus their mean regional volume across time points, isolating within-person fluctuations. Solid lines represent model-estimated associations and shaded areas indicate bootstrapped 95% confidence intervals. Only regions showing significant within-subject effects (*p* < 0.05, uncorrected) are shown.

Key findings are reported below and in Tables 2–4 and Figures 2–4, with Supplementary Tables S1–S10 providing a comprehensive account of all findings.

**Table 2:** Cross-sectional diagnostic group differences in regional brain volumes are shown for each time point.

Region	Time Point 1				Time Point 2			
	Subregion	Effect	*p*	ηp^2^	Subregion	Effect	*p*	ηp^2^
Frontal lobe GM	---	---	---	---	---	---	---	---
Basal ganglia	Left putamen	**↓**	0.040	0.067	---	---	---	---
Cerebellum GM	Vermis I–V	**↑**	0.046	0.063	---	---	---	---
Cerebellum WM	Left lobule I–V	**↓**	**0.004**	0.126	Left Lobule I–V	**↓**	0.040	0.239
	Right lobule I–V	**↓**	**0.017**	0.090				
	Left lobule VIII	**↓**	**0.006**	0.119				
	Right lobule VIII	**↓**	**0.002**	0.145				
	Right lobule IX	**↓**	0.044	0.065				
	Left lobule X	**↓**	**0.002**	0.143				
	Right lobule X	**↓**	**< 0.001**	0.185				
	Vermis I–V	**↓**	**0.010**	0.104	Vermis I–V	**↓**	0.017	0.306
	Vermis VI–VII	**↓**	**0.009**	0.107				
	Vermis VIII–X	**↓**	**< 0.001**	0.179				

Arrows indicate whether volumes were higher (↑) or lower (↓) in the pCMS group relative to the TD group. Uncorrected *p*-values are reported, with effect sizes presented as partial eta squared (ηp^2^). Bolded *p*-values indicate significance after FDR correction.

**Table 3:** Cross-sectional associations between regional brain volumes and the SSS Motor Scale are shown for each time point.

Region	Time point 1				Time point 2			
	Subregion	Effect	*p*	*r*	Subregion	Effect	*p*	*r*
Frontal Lobe GM	Left mPFC	negative	0.019	−0.432	---	---	---	---
Basal Ganglia	---	---	---	---	---	---	---	---
Cerebellum GM	Right lobule I–V	positive	0.024	0.419	---	---	---	---
Cerebellum WM	Right CrusII/VIIB	positive	0.021	0.427	Right lobule VIII	positive	0.037	0.738
					Left lobule IX	positive	0.015	0.811
					Right lobule IX	positive	0.021	0.786

Uncorrected *p*-values and Pearson correlation coefficients (r) are reported. “---“ indicates that no significant correlations were observed for that region at the given time point.

**Table 4:** Linear mixed-effects model estimates of within-subject associations between regional brain volume deviation and SSS Motor Score.

Within-subject effects (Volume deviation)						
Region	Subregion	β	SE	95% CI	*t*	*p*
Frontal lobe GM	Right DLPFC	0.0022	0.001	[0.0011, 0.0034]	3.73	0.012
	Right inferolateral PFC	0.0032	0.001	[0.0017, 0.0047]	4.26	0.005
	Right lateral OFC	0.0039	0.002	[0.0006, 0.0069]	2.53	0.031
	Right lateral premotor	0.0057	0.002	[0.002, 0.0094]	3.21	0.012
	Left primary motor	0.0040	0.001	[0.0014, 0.0067]	3.20	0.013
	Right primary motor	0.0032	0.001	[0.0006, 0.0059]	2.52	0.038
Basal Ganglia	---	---	---	---	---	---
Cerebellum GM	---	---	---	---	---	---
Cerebellum WM	---	---	---	---	---	---

Positive β coefficients indicate that increases in regional brain volume relative to an individual's mean volume across time points were associated with higher motor severity. Volume deviation was defined as the difference between the regional volume at each time point and the participant-specific mean regional volume, isolating within-person variation over time.

### 3.1 Cross-sectional effect of diagnosis on brain volume

#### 3.1.1 Frontal cortical regions

At Time Point 1 and Time Point 2, no significant effects of diagnosis were observed for any of the frontal regions.

#### 3.1.2 Basal ganglia regions

At Time Point 1, the general linear models revealed smaller left putamen volumes in the pCMS group compared to TD controls (*p* = 0.040, with a medium effect size of $\eta_{p}^{2}\;=$ 0.067). This effect did not survive FDR correction. No significant effects of diagnosis were observed for any of the basal ganglia ROIs at Time Point 2.

#### 3.1.3 Cerebellar regions

##### 3.1.3.1 Gray matter

At Time Point 1, the general linear models revealed larger gray matter volumes in the pCMS group compared to TD controls in vermis region I-V (*p* = 0.046, $\eta_{p}^{2}\;=$ 0.063); however, this effect did not survive FDR correction. No other cerebellar gray matter ROIs were significant in the Time Point 1 cohort.

At Time Point 2, no significant effects of diagnosis were observed for any of the cerebellar gray matter regions.

##### 3.1.3.2 White matter

At Time Point 1, the general linear models revealed smaller white matter volumes in the pCMS group compared to TD controls in right lobule I-V (*p* = 0.017 surviving FDR correction with a medium effect size of $\eta_{p}^{2}\;=$ 0.090), left lobule I-V (*p* = 0.004 surviving FDR correction with a medium-large effect size of $\eta_{p}^{2}$ = 0.126), right lobule VIII (*p* = 0.002 surviving FDR correction with a large effect size of $\eta_{p}^{2}\;=$ 0.145), left lobule VIII (*p* = 0.006 surviving FDR correction with a medium-large effect size of $\eta_{p}^{2}$ = 0.119), right lobule IX (*p* = 0.044 not surviving FDR correction with a medium effect size of $\eta_{p}^{2}\;=$ 0.065), right lobule X (*p* < 0.001 surviving FDR corrections with a large effect size of $\eta_{p}^{2}\;=$ 0.185), left lobule X (*p* = 0.002 surviving FDR correction with a large effect size of $\eta_{p}^{2}$ = 0.143), vermis I–V (*p* = 0.01 surviving FDR correction with a medium-large effect size of $\eta_{p}^{2}\;=$ 0.104), vermis VI-VII (*p* = 0.009 surviving FDR correction with a medium-large effect size of $\eta_{p}^{2}\;=$ 0.107), and vermis VIII-X (*p* < 0.001 surviving FDR correction with a large effect size of $\eta_{p}^{2}\;=$ 0.179).

At Time Point 2, the general linear models revealed smaller white matter volumes in the pCMS group compared to TD controls in left lobule I-V (*p* = 0.040 not surviving FDR correction with a large effect size of $\eta_{p}^{2}$ = 0.239) and vermis I–V (*p* = 0.017 not surviving FDR corrections with a large effect size of $\eta_{p}^{2}$ = 0.306).

### 3.2 pCMS brain volume and SSS correlations

#### 3.2.1 Frontal cortical regions

At Time Point 1, smaller gray matter volumes in the left mPFC were associated with higher scores (greater severity) on the SSS Motor Score (*r* = −0.432, *p* = 0.019 but does not survive FDR corrections) among the pCMS group.

At Time Point 2, no significant associations were observed between any frontal cortical regions and SSS Motor Scores.

#### 3.2.2 Basal ganglia regions

No significant associations were observed between any basal ganglia volumes and SSS Motor Scores at either time point.

#### 3.2.3 Cerebellar regions

##### 3.2.3.1 Gray matter

At Time Point 1, larger gray matter volumes in right lobule I-V were associated with higher (greater severity) SSS Motor Scores (*r* = 0.419, *p* = 0.024 but does not survive FDR correction).

At Time Point 2, no significant correlations were observed between any of the cerebellar gray matter volumes and SSS Motor Scores among the pCMS group.

##### 3.2.3.2 White matter

At Time Point 1, larger white matter volumes in right crus II/VIIB were associated with higher (greater severity) SSS Motor Scores (*r* = 0.427, *p* = 0.021 but does not survive FDR correction), among the pCMS group.

At Time Point 2, larger white matter volumes were associated with higher (greater severity) SSS Motor Scores in right lobule VIII (*r* = 0.738, *p* = 0.037), right lobule IX (*r* = 0.786, *p* = 0.037), and left lobule IX (*r* = 0.811, *p* = 0.021) among the pCMS group but effects did not survive FDR correction.

### 3.3 Developmental changes in pCMS brain volume and stereotypy severity (T1-T2)

#### 3.3.1 Frontal cortical regions

Linear mixed-effects models revealed that within-subject increases in frontal lobe gray matter volumes were associated with higher SSS Motor Scores. Notably, significant effects were observed in the left (β = 0.0040, SE = 0.001, *t* = 3.20, *p* = 0.013) and right (β = 0.0032, SE = 0.001, *t* = 2.52, *p* = 0.038) primary motor cortices, right DLPFC (β = 0.0022, SE = 0.001, *t* = 3.73, *p* = 0.012), right inferolateral PFC (β = 0.0032, SE = 0.001, *t* = 4.26, *p* = 0.005), right lateral OFC (β = 0.0039, SE = 0.002, *t* = 2.53, *p* = 0.031), and right lateral premotor cortex (β = 0.0057, SE = 0.002, *t* = 3.21, *p* = 0.012). The results suggest that changes in frontal lobe volumes may be related to concurrent fluctuations in stereotypy symptom severity. None of the findings survived FDR correction.

#### 3.3.2 Basal ganglia regions

Linear mixed-effects models revealed no significant within-subject associations between regional basal ganglia volumes and SSS Motor Scores.

#### 3.3.3 Cerebellar regions

Linear mixed-effects models revealed no significant within-subject associations between regional cerebellar gray or white matter volumes and SSS Motor Scores.

## 4 Discussion

The goal of this study is to use brain volumetric MRI analyses to better understand the underlying pathophysiology of pCMS. More specifically, the objective being to further clarify prior suggestions of alterations within the frontal cortex, basal ganglia, and cerebellum (12–14) by utilizing a larger sample size, examining brain structure with a higher magnetic field (3T), controlling for possible multiple hypothesis testing errors (FDR), and conducting both cross-sectional and longitudinal analyses in affected children and adolescents/young adults. As illustrated in Figure 1, each of these selected brain regions are components of, or significant contributors to, the CBGTC circuit. Readers should, however, be aware that any volumetric alterations identified in this report can only be considered pCMS associated findings, and subsequent discussions regarding their potential effects are necessarily hypothesis driven. More specifically, a formal inference of circuit imbalance or normalization requires evidence derived from measures of structural and functional connectivity, diffusion-based imaging, electrophysiological recordings or other physiological approaches capable of assessing communication among network components. In the absence of such measures, the present findings should not be interpreted as direct evidence of circuit dysfunction, but rather as observations that may inform future mechanistic hypotheses regarding circuit involvement.

### 4.1 Basal ganglia involvement

The basal ganglia include the striatum [caudate, putamen, nucleus accumbens], the subthalamic nucleus (STN), the globus pallidus interna (GPi) and externa (GPe), ventral pallidum (VP), and the substantia nigra (pars compacta, SNpc, and reticulata). The striatum and STN receive most of their inputs from the cerebral cortex and the thalamus. Outputs from the basal ganglia primarily arise from the GPi, VP, and SNpc with major projections to the thalamus and subsequently the motor cortex. The basal ganglia has been a strong potential candidate in the etiology of motor stereotypies based upon human fetal studies showing disproportional frontal cortical connections to the basal ganglia (31), its role in the selection and implementation of movements (32), involvement in goal-directed and habitual movements (11, 33), and a widespread role in other movement disorders (34).

In the current study, at the childhood baseline (Time Point 1), analyses across all basal ganglia regions showed only a significant group difference in the left putamen that did not survive FDR correction. No significant group differences were identified in the globus pallidus or caudate volume. Further, there was no significant statistical association between a basal ganglia region and stereotypy severity. At Time Point 2, in the adolescent/young adult pCMS population, results showed no basal ganglia differences compared to TD controls and no basal ganglia–severity associations. Our findings of smaller childhood putamen volume in children with pCMS, prior to FDR correction for multiple comparisons, are similar to results published in a prior study (also with uncorrected comparisons) in 19 children with pCMS, ages 8–12 years. In this earlier 3.0T protocol, results showed significant reductions in the right and a trend for reduction in the left putamen volume with no significant differences in the caudate (13). In a separate earlier volumetric pCMS study using a 1.5T scanner in 6, age 9–11-year-old males, volumetric reductions were reported in the caudate plus a widespread frontal white matter loss ^2^. Neither of these two earlier studies, however, showed a significant association between anatomical changes and stereotypy severity.

In summary, the current volumetric data, while showing some early marginal focal alterations, does not support a direct severity-linked volumetric basal ganglia involvement in pCMS. Pathophysiologically, however, the available data does not completely negate the possibility that developmentally early striatal/pallidal differences could subsequently influence the development and maturation of cortical-striatal circuits that balance goal-directed and habitual movement control. The latter speculation supported by findings from a MRI functional connectivity study showing reduced prefrontal cortical-striatal coupling in pCMS children (8–12 years of age) (14). Clearly additional investigations are required to determine whether early striatal changes confer risk for later alterations in the trajectory and maturation of frontal-striatal control systems and/or pallidal output.

### 4.2 Frontal cortical alterations

The prefrontal cortex, located on the anterior portion of the frontal lobe, is anatomically, functionally, and computationally complex (35–39). This region starts to develop before birth, progresses slowly throughout childhood, and finally completes its development process in late adolescence. Anatomically, it contains several subregions, each with differing projections, and its functional activities include motor control, planning, decision making, working memory, sensory attention, abstractions, etc. The prefrontal cortices are key nodes in top-down control networks, integrating motor, emotional, and reward-related information to shape behavior.

The rationale for pursuing frontal cortical volumetric measurements in this study was 2-fold. The first, being to reinvestigate a prior imaging study in six males with pCMS that identified volumetric reductions in frontal white matter that was disproportionate to total cerebral white matter (12). In the current study at Time Point 1 (pre-pubertal, school-age children), there were no significant group differences across frontal cortical ROIs. A smaller volume in the left medial prefrontal cortex was associated with increased stereotypy severity but did not survive FDR correction. At Time Point 2 (post-pubertal, adolescents/young adults), there were no significant associations between any frontal cortical regions and motor stereotypy severity.

A second reason for investigating cortical volumetric measurements was to further investigate whether an evolving frontal cortical pattern could explain a documented significant reduction in the intensity of motor stereotypies in adolescents with pCMS (1). The latter hypothesis being generated, in part, from longitudinal studies in autistic spectrum disorder children that showed age-related maturational cortical thinning in prefrontal and parietal regions is associated with changes in repetitive behaviors over time (40, 41). To further explore this hypothesis, linear mixed-effects models were used to evaluate associations between stereotypy severity and regional brain volumes in our longitudinal cohort of pCMS subjects. Of interest, results identified multiple significant associations between within-subject increases in frontal lobe gray matter volumes (primary motor cortex, right DLPFC, right inferolateral PFC, right lateral OFC, and right lateral premotor cortex) and higher SSS Motor Scores (increased stereotypy severity). Due to the relatively small number of participants, however, this finding must be considered preliminary and requires replication in larger cohorts. If confirmed, however, their emergence in relation to longitudinal changes in stereotypy symptoms, but not diagnostic status, likely underscores their role in modulating habitual motor output as children mature, particularly given that thinning of frontal cortex (and resulting decrease in volume) through adolescence reflects typical maturation (11).

### 4.3 Cerebellar contributions

Anatomically, the cerebellum and basal ganglia participate via densely interconnected motor networks with the cerebral cortex [Figure 1; (11, 42)]. The cerebellum receives input from both the cortex (via the pontine nucleus and olivocerebellar pathways) and the basal ganglia (from the STN, via transmission through the pedunculopontine tegmental nucleus (PPTg). The cerebellum sends excitatory projections directly to the thalamus, which in turn relays signals to both motor and associative striatal territories (43, 44), and has direct contact with the amygdala, dorsal raphe nucleus (DRN), and ventral tegmental area.

Several lines of neuroanatomical evidence have previously suggested a role for the cerebellum in the pathophysiology of motor stereotypies. For example, cerebellar malformations (e.g., rhombencephalosynapsis) and early cerebellar developmental injuries are associated with childhood repetitive behaviors (14, 45, 46), and functional imaging studies demonstrate cerebellar activation during motor tasks and repetitive behaviors (47–49). In a prior pCMS study, involving 20 children (ages 8–12 years; 12 boys and 8 girls), there was a statistically significant reduction in white matter volume in posterior cerebellar lobules VI–VII, which negatively correlated with motor control, and an 8% increase in anterior vermis gray matter, which positively correlated with stereotypy severity (14).

In this protocol, cerebellar findings were generally more robust than either basal ganglia or frontal cortical findings. At Time Point 1 for gray matter, the pCMS group showed increased vermal I-V gray matter volume that disappeared with FDR correction, and for white matter, there were reductions surviving FDR correction in lobules I–V (bilateral), lobule VI (bilateral), lobule VIII (bilateral), lobule X (bilateral), and vermal regions (I–X). At Time Point 2, the pCMS group showed several smaller white matter volumes but none persisted after FDR correction. In terms of clinical relevance, larger average posterior and inferior cerebellar volumes in pCMS subjects did initially correlate with higher SSS Motor scores; however, it is worth noting that this finding did not survive FDR correction.

Findings in the current study and recent publications provide several interesting facts, but not definitive answers regarding cerebellar anatomy and its potential association with motor stereotypies. The identified gray/white matter dissociation in the current protocol was not diffuse, but regionally specific, involving either sensorimotor cerebellar territories (anterior vermis and lobule VIII), which map onto cerebello-thalamo-cortical motor loops involved in motor execution and coordination (50) or non-motor functional domains (posterior vermis, crus I, lobule X), which have been implicated in affective, cognitive, and vestibular functions (50). The anterior vermis and lobule VIII, have been linked to stereotyped and repetitive motor behaviors in both clinical and animal models (14, 49, 50). Excitatory connections from the dentate, interposed, and fastigial nuclei of the cerebellum have been shown to rapidly modulate activity in the substantia nigra (51, 52). Further, the cerebellum generates variable dysfunctional spike patterns that might explain their ability to cause a diverse pattern of motor abnormalities (53). Rodent studies have documented functional connectivity between the cerebellum and the medial prefrontal cortex (mPFC) via the ventromedial thalamus and shown that the mPFC mediates cerebellum-regulated social and repetitive/inflexible behaviors (54). Lastly, disruptions in connectivity between the cerebellum and mPFC have been identified in multiple mouse models of ASD-linked genes and in individuals with ASD (54), and distinct cerebellar networks have been suggested to underpin clinical improvement in Tourette syndrome (55).

In summary, current findings support, but do not definitively confirm, that the cerebellar sensorimotor circuitry is a pathophysiological process linked to stereotypy symptom expression and progression over time. Nevertheless, the latter hypothesis is gaining further relevance based on preclinical studies delineating a cerebello-thalamo-prefrontal circuit; extending from right crus I and posterior vermis through the cerebellar nuclei and ventromedial thalamus to the mPFC where modulation of cerebellar output induces repetitive behaviors and social deficits in rodent models (54).

## 5 Limitations and future directions

Limitations include changes in head coil used between Time Point 1 (8 channel) and Time Point 2 (32 channel), smaller Time Point 2 and longitudinal sample sizes, various study variabilities, and reliance on volume measurements.

As discussed in detail in the Methods, MRI data was acquired using different head coils at Time Points 1 and 2. Although all analyses were statistically controlled for head coil type, residual confounding may persist due to the uneven distribution of coil between diagnostic groups (significantly different in Time Point 1) and the complete confounding of coil type between time points in the pCMS group. As a result, findings could partially reflect acquisition differences rather than purely biological difference or change and should be interpreted with this limitation in mind.

Another limitation is that the Time Point 2 sample was not balanced with respect to sex across groups. Based on established sex differences in neurodevelopment and brain volume measures, unequal sex distribution could influence the observed relationships. Future studies with sex-balanced or sex-stratified samples will be important to clarify whether these findings generalize across sexes.

The longitudinal design utilized in this study is a strength; however, more than two time points are needed to model non-linear developmental trajectories and to separate age-related change from cohort effects. The later, representing an expansion of the current approach rather than a negation of the present findings.

Additional sources of variability include cohort differences at Time Point 2 (new controls), attrition/return bias, potential differences in the optimal informant for symptom severity in older adolescents (parent vs. self-report), the potential impact of comorbidities on both imaging findings and severity scores, and technical factors such as head coil differences (8 vs. 32 channel). Since some cerebellar subdivisions are small, they may be more susceptible to segmentation noise. Further, hemisphere-collapsed analyses may mask meaningful lateralization.

Other essential connections, not measured in this protocol, could be contributing to pCMS neurobiology. For example, the ventromedial thalamus processes inputs from the cortex that are important for generating persist activity and receives inputs from SNPR and cerebellum that are forwarded to the prefrontal cortex (56). In addition, the ventral striatum integrates limbic input from the amygdala and hippocampus and supports reward, emotion, vigor, attention, and autonomic regulation; this circuitry could modulate stereotypy expression without clear volumetric signatures in the regions analyzed here.

Another important limitation is the absence of neuropsychological or behavioral measures. Aspects that would allow a more direct evaluation of the functional significance of structural alterations and their relationship to symptom improvement. Future investigations should also integrate diffusion and spectroscopy to distinguish pruning from myelination or connectivity shifts. Lastly, longitudinal functional connectivity analyses are required to map the dynamic interactions between structural nodes and possibly validate a circuit-normalization hypothesis.

## 6 Conclusion

This study was designed to further clarify our understanding of previously reported volumetric abnormalities involving the basal ganglia, cerebellum, and frontal cortex in children with primary complex motor stereotypies (pCMS). Methodologically, two cross-sectional analyses in different age groups s were used to compare volumetric differences between affected and typically developing individuals. In addition, a separate two-point longitudinal analysis was conducted to examine whether changes in regional brain volumes were associated with changes in stereotypy severity. Results of these analyses continue to suggest that pCMS may be associated with regionally specific and developmentally dynamic structural differences, rather than a single neuroanatomical abnormality. Of the studied brain regions, surprisingly current data supports a likely etiological involvement of the cerebellum in the pathophysiology of primary CMS. In addition, preliminary results suggest a positive association between maturational increases in frontal lobe gray matter volumes and an increase in stereotypy severity in adolescents and young adults. Thus, in the absence of definitive evidence implicating a single abnormal brain region and given that existing findings continue to suggest that pCMS may be best conceptualized as a circuit-level disorder, future longitudinal imaging studies are recommended. More specifically, in individuals with pCMS, investigations should incorporate network-based analyses, including measures of structural and functional connectivity, to directly examine circuit-level organization and determine whether alterations in network interactions contribute to symptom expression and developmental change.

## Data Availability

The raw data supporting the conclusions of this article will be made available by the authors, without undue reservation.
