# Supplementary material for: Volumetric brain alterations in children with primary complex motor stereotypies: a baseline and longitudinal report
Source: Front Neurol. 2026 Jul 22;17:1800595. doi: 10.3389/fneur.2026.1800595 (PMC13439610; doi:10.3389/fneur.2026.1800595)
Supplement: Supplementary file 1 [file Supplementary_file_1.docx]

**Supplementary material**

**Supplementary Table 1: Effect of Dx on ROI for Frontal Cortical Regions**

|  | **Time Point 1** | | | | |
| --- | --- | --- | --- | --- | --- |
|  | **Region** | **pCMS**  **Mean** | **TD**  **Mean** | **Uncorrected**  **p-value** | **Partial Eta Sq** |
|  | Left Dorsolateral PFC | 21973.464 | 21994.142 | 0.972 | <0.000 |
|  | Right Dorsolateral PFC | 23334.725 | 23364.639 | 0.958 | <0.000 |
|  | Left Medial PFC | 16335.716 | 16518.436 | 0.684 | 0.003 |
|  | Right Medial PFC | 17373.142 | 17548.282 | 0.652 | 0.003 |
|  | Left Inferolateral PFC | 12450.671 | 12333.208 | 0.780 | 0.001 |
|  | Right Inferolateral PFC | 12931.462 | 13117.508 | 0.644 | 0.004 |
|  | Left Lateral OFC | 13643.371 | 14132.932 | 0.118 | 0.040 |
|  | Right Lateral OFC | 11364.564 | 11433.678 | 0.763 | 0.001 |
|  | Left Medial OFC | 4361.217 | 4307.207 | 0.688 | 0.003 |
| Right Medial OFC | | 5359.189 | 5428.114 | 0.659 | 0.003 |
|  | Left Supplementary Motor | 5753.953 | 5748.259 | 0.975 | <0.000 |
|  | Right Supplementary Motor | 5043.386 | 5178.584 | 0.393 | 0.012 |
|  | Left Frontal Eye Field | 2701.950 | 2602.807 | 0.436 | 0.010 |
|  | Right Frontal Eye Field | 2197.498 | 2248.169 | 0.664 | 0.003 |
|  | Left Lateral Premotor | 7137.540 | 6938.399 | 0.456 | 0.009 |
|  | Right Lateral Premotor | 7011.697 | 7197.697 | 0.418 | 0.011 |
|  | Left Primary Motor | 12919.293 | 12665.828 | 0.432 | 0.010 |
|  | Right Primary Motor | 13747.815 | 14026.548 | 0.335 | 0.015 |
|  | Left Anterior Cingulate | 7781.246 | 7909.208 | 0.636 | 0.004 |
|  | Right Anterior Cingulate | 7056.942 | 6986.119 | 0.720 | 0.002 |
|  | **Time Point 2** | | | | |
|  | **Region** | **pCMS**  **Mean** | **TD**  **Mean** | **Uncorrected**  **p-value** | **Partial Eta Sq** |
|  | Left Dorsolateral PFC | 18317.081 | 19145.448 | 0.369 | 0.051 |
|  | Right Dorsolateral PFC | 18872.286 | 18642.951 | 0.796 | 0.004 |
|  | Left Medial OFC | 4190.864 | 4163.514 | 0.921 | 0.001 |
|  | Right Medial OFC | 4876.324 | 4763.124 | 0.722 | 0.008 |
|  | Left Inferolateral PFC | 10015.594 | 9915.653 | 0.904 | 0.001 |
|  | Right Inferolateral PFC | 10493.548 | 10432.158 | 0.935 | <0.000 |
|  | Left Lateral OFC | 12614.646 | 12231.028 | 0.563 | 0.021 |
|  | Right Lateral OFC | 10480.305 | 10234.593 | 0.637 | 0.014 |
|  | Left Medial PFC | 14346.785 | 13674.620 | 0.335 | 0.058 |
|  | Right Medial PFC | 15421.770 | 15267.529 | 0.849 | 0.002 |
|  | Left Supplementary Motor | 5381.101 | 5186.087 | 0.592 | 0.018 |
|  | Right Supplementary Motor | 5148.313 | 5033.583 | 0.803 | 0.004 |
|  | Left Frontal Eye Field | 2739.878 | 2765.274 | 0.905 | 0.001 |
|  | Right Frontal Eye Field | 2147.048 | 2427.047 | 0.285 | 0.071 |
|  | Left Lateral Premotor | 6552.910 | 6847.008 | 0.542 | 0.024 |
|  | Right Lateral Premotor | 6424.436 | 6736.196 | 0.311 | 0.064 |
|  | Left Primary Motor | 12647.963 | 12570.494 | 0.894 | 0.001 |
|  | Right Primary Motor | 13456.801 | 13317.599 | 0.833 | 0.003 |
|  | Left Anterior Cingulate | 6648.610 | 6947.853 | 0.441 | 0.038 |
|  | Right Anterior Cingulate | 6526.992 | 6182.345 | 0.200 | 0.100 |

pCMS = primary complex motor stereotypy; TD = typically developing Uncorrected significant *p-values* are indicated in bold. No effects survive FDR correction for multiple comparisons at α=0.05.

**Supplementary Table 2: Effect of Dx on ROI for Subcortical Regions, Time 1 and Time 2**

| **Time Point 1** | | | | |
| --- | --- | --- | --- | --- |
| **Region** | **pCMS**  **Mean** | **TD**  **Mean** | **Uncorrected**  **p-value** | **Partial**  **Eta Sq** |
| Left Caudate | 4102.635 | 4190.725 | 0.338 | 0.015 |
| Right Caudate | 4179.716 | 4233.794 | 0.540 | 0.006 |
| Left Putamen | 4734.771 | 4903.364 | **0.040** | 0.067 |
| Right Putamen | 4699.340 | 4809.713 | 0.152 | 0.033 |
| Left Globus Pallidus | 1717.871 | 1778.689 | 0.063 | 0.056 |
| Right Globus Pallidus | 1595.317 | 1652.245 | 0.054 | 0.059 |
| **Time Point 2** | | | | |
| **Region** | **pCMS**  **Mean** | **TD**  **Mean** | **Uncorrected**  **p-value** | **Partial**  **Eta Sq** |
| Left Caudate | 4323.984 | 4300.271 | 0.928 | 0.001 |
| Right Caudate | 4204.758 | 4401.408 | 0.485 | 0.031 |
| Left Putamen | 121.090 | 144.526 | 0.734 | 0.007 |
| Right Putamen | 4847.947 | 4836.938 | 0.958 | <0.000 |
| Left Globus Pallidus | 1767.120 | 1812.177 | 0.506 | 0.028 |
| Right Globus Pallidus | 1651.899 | 1691.359 | 0.441 | 0.037 |

pCMS = primary complex motor stereotypy; TD = typically developing. Uncorrected significant *p-values* are indicated in bold. No effects survive FDR correction for multiple comparisons at α=0.05.

**Supplementary Table 3: Effect of Dx on ROI for Cerebellar Regions (gray and white matter), Time 1 and Time 2**

**Time Point 1**

|  | **Gray Matter** | | | | **White Matter** | | | |
| --- | --- | --- | --- | --- | --- | --- | --- | --- |
| **Region** | **pCMS**  **Mean** | **TD**  **Mean** | **Uncorrected**  **p-value** | **Partial**  **Eta Sq** | **pCMS**  **Mean** | **TD**  **Mean** | **Uncorrected**  **p-value** | **Partial**  **Eta Sq** |
| Right Lobule I-V | 4886.561 | 4695.105 | 0.269 | 0.020 | 602.014 | 711.571 | **0.017*** | 0.090 |
| Left Lobule I-V | 5244.973 | 5174.305 | 0.716 | 0.002 | 547.349 | 663.670 | **0.004*** | 0.126 |
| Right Lobule VI | 9175.389 | 8964.576 | 0.535 | 0.006 | 790.638 | 875.973 | 0.066 | 0.055 |
| Left Lobule VI | 9572.133 | 9273.888 | 0.345 | 0.015 | 667.863 | 732.254 | 0.088 | 0.047 |
| Right Crus I | 12280.269 | 11887.415 | 0.319 | 0.016 | 1837.266 | 1829.024 | 0.931 | <0.000 |
| Left Crus I | 12371.370 | 12015.221 | 0.378 | 0.013 | 1448.965 | 1504.274 | 0.483 | 0.008 |
| Right Crus II/VIIB | 11672.451 | 11320.961 | 0.419 | 0.011 | 2152.739 | 2241.045 | 0.557 | 0.006 |
| Left Crus II/VIIB | 12427.100 | 12179.193 | 0.565 | 0.005 | 1820.638 | 1961.798 | 0.250 | 0.022 |
| Right Lobule VIII | 8569.076 | 8637.511 | 0.836 | 0.001 | 1407.236 | 1728.739 | **0.002*** | 0.145 |
| Left Lobule VIII | 8824.614 | 8704.064 | 0.672 | 0.003 | 1271.715 | 1538.981 | **0.006*** | 0.119 |
| Right Lobule IX | 2719.787 | 2701.718 | 0.901 | <0.000 | 437.649 | 513.930 | **0.044** | 0.065 |
| Left Lobule IX | 2722.801 | 2672.474 | 0.703 | 0.002 | 420.050 | 465.640 | 0.190 | 0.028 |
| Right Lobule X | 368.473 | 373.920 | 0.778 | 0.001 | 59.780 | 89.468 | **<0.001*** | 0.185 |
| Left Lobule X | 383.029 | 412.022 | 0.156 | 0.033 | 55.587 | 75.132 | **0.002*** | 0.143 |
| Vermis I-V | 1841.079 | 1739.114 | **0.046** | 0.063 | 229.351 | 290.025 | **0.01*** | 0.104 |
| Vermis VI-VII | 1221.923 | 1217.750 | 0.914 | <0.000 | 115.676 | 140.042 | **0.009*** | 0.107 |
| Vermis VIII-X | 1989.763 | 1965.444 | 0.691 | 0.003 | 109.277 | 163.043 | **<0.001*** | 0.179 |
| Corpus Medullare | - | - | - | - | 10961.460 | 11302.743 | 0.240 | 0.023 |
|  | **Time Point 2** | | | | | | | |
|  | **Gray Matter** | | | | **White Matter** | | | |
| **Region** | **pCMS**  **Mean** | **TD**  **Mean** | **Uncorrected**  **p-value** | **Partial**  **Eta Sq** | **pCMS**  **Mean** | **TD**  **Mean** | **Uncorrected**  **p-value** | **Partial**  **Eta Sq** |
| Right Lobule I-V | 4798.822 | 4814.303 | 0.975 | <0.000 | 586.246 | 738.776 | 0.053 | 0.215 |
| Left Lobule I-V | 5296.851 | 5126.264 | 0.735 | 0.007 | 579.885 | 739.432 | **0.040** | 0.239 |
| Right Lobule VI | 9295.181 | 8956.952 | 0.660 | 0.012 | 822.656 | 820.993 | 0.984 | <0.000 |
| Left Lobule VI | 9496.798 | 9251.525 | 0.716 | 0.008 | 718.144 | 752.052 | 0.587 | 0.019 |
| Right Crus I | 12299.215 | 12491.636 | 0.810 | 0.004 | 1961.57 | 1752.558 | 0.157 | 0.121 |
| Left Crus I | 12922.240 | 12940.192 | 0.983 | <0.000 | 1742.848 | 1510.418 | 0.081 | 0.179 |
| Right Crus II/VIIB | 11496.979 | 11995.692 | 0.613 | 0.016 | 2223.256 | 2221.412 | 0.994 | <0.000 |
| Left Crus II/VIIB | 12279.486 | 12796.009 | 0.638 | 0.014 | 2195.47 | 1965.034 | 0.407 | 0.043 |
| Right Lobule VIII | 8752.863 | 8717.935 | 0.966 | <0.000 | 1610.886 | 1777.922 | 0.652 | 0.013 |
| Left Lobule VIII | 8802.952 | 8791.930 | 0.982 | <0.000 | 1468.053 | 1610.347 | 0.625 | 0.015 |
| Right Lobule IX | 2787.299 | 2544.742 | 0.487 | 0.031 | 603.790 | 530.363 | 0.429 | 0.039 |
| Left Lobule IX | 2867.023 | 2626.579 | 0.474 | 0.033 | 562.049 | 509.376 | 0.580 | 0.020 |
| Right Lobule X | 382.172 | 376.786 | 0.912 | 0.001 | 44.023 | 57.790 | 0.062 | 0.202 |
| Left Lobule X | 396.393 | 369.662 | 0.633 | 0.015 | 50.638 | 53.706 | 0.629 | 0.015 |
| Vermis I-V | 1845.594 | 1810.893 | 0.806 | 0.004 | 286.078 | 401.971 | **0.017** | 0.306 |
| Vermis VI-VII | 1235.214 | 1220.577 | 0.868 | 0.002 | 159.662 | 177.613 | 0.400 | 0.045 |
| Vermis VIII-X | 2022.614 | 2014.636 | 0.957 | <0.000 | 165.782 | 233.706 | 0.093 | 0.166 |
| Corpus Medullare | - | - | - | - | 12278.312 | 12751.078 | 0.475 | 0.032 |

pCMS = primary complex motor stereotypy; TD = typically developing. Uncorrected significant *p-values* are indicated in bold. Asterisks represent effects that survive FDR correction for multiple comparisons at α=0.05.

**Supplementary Table 4: Correlations between Frontal ROI’s and SSS scores, Time 1 and Time 2**

|  | **Time Point 1** |  |
| --- | --- | --- |
| **Region** | **Pearson Correlation Coefficient (r)** | **Uncorrected**  **p-value** |
| Left Dorsolateral PFC | -0.132 | 0.495 |
| Right Dorsolateral PFC | 0.167 | 0.386 |
| Left Medial PFC | -0.432 | **0.019** |
| Right Medial PFC | 0.103 | 0.594 |
| Left Inferolateral PFC | 0.006 | 0.977 |
| Right Inferolateral PFC | 0.012 | 0.952 |
| Left Lateral OFC | 0.209 | 0.277 |
| Right Lateral OFC | 0.239 | 0.212 |
| Left Medial OFC | 0.345 | 0.067 |
| Right Medial OFC | -0.022 | 0.909 |
| Left Supplementary Motor | 0.281 | 0.140 |
| Right Supplementary Motor | 0.319 | 0.092 |
| Left Frontal Eye Field | 0.194 | 0.313 |
| Right Frontal Eye Field | 0.331 | 0.079 |
| Left Lateral Premotor | 0.065 | 0.737 |
| Right Lateral Premotor | 0.139 | 0.471 |
| Left Primary Motor | 0.139 | 0.473 |
| Right Primary Motor | 0.275 | 0.149 |
| Left Anterior Cingulate | -0.184 | 0.339 |
| Right Anterior Cingulate | 0.244 | 0.202 |
|  | **Time Point 2** |  |
| **Region** | **Pearson Correlation Coefficient (r)** | **Uncorrected**  **p-value** |
| Left Dorsolateral PFC | 0.184 | 0.663 |
| Right Dorsolateral PFC | 0.554 | 0.154 |
| Left Medial OFC | -0.224 | 0.594 |
| Right Medial OFC | 0.127 | 0.764 |
| Left Inferolateral PFC | 0.043 | 0.920 |
| Right Inferolateral PFC | 0.028 | 0.947 |
| Left Lateral OFC | 0.141 | 0.739 |
| Right Lateral OFC | 0.074 | 0.861 |
| Left Medial PFC | -0.277 | 0.507 |
| Right Medial PFC | -0.415 | 0.306 |
| Left Supplementary Motor | -0.467 | 0.243 |
| Right Supplementary Motor | -0.341 | 0.409 |
| Left Frontal Eye Field | -0.050 | 0.906 |
| Right Frontal Eye Field | 0.307 | 0.460 |
| Left Lateral Premotor | 0.091 | 0.831 |
| Right Lateral Premotor | 0.054 | 0.898 |
| Right Primary Motor | -0.244 | 0.560 |
| Left Primary Motor | 0.263 | 0.529 |
| Left Anterior Cingulate | 0.068 | 0.873 |
| Right Anterior Cingulate | 0.490 | 0.217 |

Uncorrected significant *p-values* are indicated in bold. No effects survive FDR correction for multiple comparisons at α=0.05.

**Supplementary Table 5: Correlations between Subcortical ROI’s and SSS scores, Time 1 and Time 2**

|  | **Time Point 1** |  |
| --- | --- | --- |
| **Region** | **Pearson Correlation Coefficient (r)** | **Uncorrected**  **p-value** |
| Left Caudate | 0.088 | 0.648 |
| Right Caudate | 0.131 | 0.497 |
| Left Putamen | 0.132 | 0.494 |
| Right Putamen | 0.141 | 0.467 |
| Left Globus Pallidus | 0.090 | 0.642 |
| Right Globus Pallidus | 0.192 | 0.317 |
|  | **Time Point 2** |  |
| **Region** | **Pearson Correlation Coefficient (r)** | **Uncorrected**  **p-value** |
| Left Caudate | 0.343 | 0.406 |
| Right Caudate | 0.295 | 0.479 |
| Left Putamen | 0.202 | 0.632 |
| Right Putamen | 0.120 | 0.777 |
| Left Globus Pallidus | 0.244 | 0.560 |
| Right Globus Pallidus | 0.231 | 0.581 |

Uncorrected significant *p-values* are indicated in bold. No effects survive FDR correction for multiple comparisons at α=0.05.

**Supplementary Table 6: Correlations between Cerebellar ROI’s and SSS scores, Time 1 and Time 2**

|  | **Time Point 1** | | | | |
| --- | --- | --- | --- | --- | --- |
|  | | **Gray Matter** | | **White Matter** | |
| **Region** | | **Pearson Correlation Coefficient (r)** | **Uncorrected**  **p-value** | **Pearson Correlation Coefficient (r)** | **Uncorrected**  **p-value** |
| Right Lobule I-V | | 0.419 | **0.024** | 0.037 | 0.850 |
| Left Lobule I-V | | 0.252 | 0.187 | -0.075 | 0.698 |
| Right Lobule VI | | 0.192 | 0.317 | 0.138 | 0.474 |
| Left Lobule VI | | 0.365 | 0.052 | 0.206 | 0.284 |
| Right Crus I | | -0.046 | 0.814 | 0.139 | 0.472 |
| Left Crus I | | 0.021 | 0.913 | -0.032 | 0.870 |
| Right Crus II/VIIB | | 0.357 | 0.058 | 0.427 | **0.021** |
| Left Crus II/VIIB | | 0.330 | 0.080 | 0.204 | 0.288 |
| Right Lobule VIII | | -0.203 | 0.292 | 0.026 | 0.895 |
| Left Lobule VIII | | 0.057 | 0.769 | 0.227 | 0.237 |
| Right Lobule IX | | 0.043 | 0.826 | 0.164 | 0.395 |
| Left Lobule IX | | 0.069 | 0.722 | 0.281 | 0.140 |
| Right Lobule X | | -0.051 | 0.792 | -0.131 | 0.500 |
| Left Lobule X | | 0.111 | 0.567 | 0.043 | 0.824 |
| Vermis I-V | | 0.309 | 0.102 | -0.117 | 0.547 |
| Vermis VI-VII | | 0.195 | 0.311 | 0.031 | 0.871 |
| Vermis VIII-X | | -0.003 | 0.987 | -0.042 | 0.827 |
| Corpus Medullare | | - | - | 0.196 | 0.309 |
|  | | **Time Point 2** | | | |
|  | | **Gray Matter** | | **White Matter** | |
| **Region** | | **Pearson Correlation Coefficient (r)** | **Uncorrected**  **p-value** | **Pearson Correlation Coefficient (r)** | **Uncorrected**  **p-value** |
| Right Lobule I-V | | -0.124 | 0.770 | 0.442 | 0.273 |
| Left Lobule I-V | | -0.383 | 0.349 | -0.178 | 0.674 |
| Right Lobule VI | | -0.189 | 0.655 | -0.273 | 0.514 |
| Left Lobule VI | | -0.009 | 0.984 | 0.384 | 0.347 |
| Right Crus I | | -0.173 | 0.682 | -0.037 | 0.931 |
| Left Crus I | | -0.334 | 0.419 | 0.052 | 0.903 |
| Right Crus II/VIIB | | -0.385 | 0.347 | 0.217 | 0.606 |
| Left Crus II/VIIB | | 0.029 | 0.946 | 0.399 | 0.327 |
| Right Lobule VIII | | 0.543 | 0.164 | 0.738 | **0.037** |
| Left Lobule VIII | | 0.130 | 0.758 | 0.652 | 0.080 |
| Right Lobule IX | | 0.203 | 0.629 | 0.786 | **0.021** |
| Left Lobule IX | | 0.175 | 0.678 | 0.811 | **0.015** |
| Right Lobule X | | 0.019 | 0.964 | 0.152 | 0.720 |
| Left Lobule X | | 0.056 | 0.895 | 0.318 | 0.443 |
| Vermis I-V | | 0.369 | 0.369 | 0.389 | 0.341 |
| Vermis VI-VII | | 0.627 | 0.096 | 0.167 | 0.693 |
| Vermis VIII-X | | -0.208 | 0.621 | 0.394 | 0.334 |
| Corpus Medullare | | - | - | 0.503 | 0.204 |

Uncorrected significant *p-values* are indicated in bold. No effects survive FDR correction for multiple comparisons at α=0.05.
